# Specialized Dendritic Cells Mediating Peripheral Tolerance to Intestinal Antigens

**DOI:** 10.1111/imr.70082

**Published:** 2025-12-02

**Authors:** Liuhui Fu, Dan R. Littman

**Affiliations:** ^1^ Department of Cell Biology New York University School of Medicine New York New York USA; ^2^ Howard Hughes Medical Institute New York New York USA

**Keywords:** AIRE, CCR7, cDC1, integrin α_v_β_8_, tolerizing dendritic cell (tolDC), Treg therapy

## Abstract

The immune system is tasked with mounting effective responses to pathogens while preventing inflammation triggered by innocuous antigens, including those derived from self, food, and commensal microbes. This balance is especially critical in the intestine, where dietary and microbial antigens are constantly encountered. Peripherally induced regulatory T cells (pTreg or iTreg) play a key role in suppressing inappropriate immune activation and maintaining gut homeostasis. Elucidating how pTreg cells are generated along the gastrointestinal tract is therefore critical to understanding peripheral tolerance. Recent studies have revealed that intestinal antigen‐specific pTreg cell differentiation is induced by a distinct lineage of antigen‐presenting cells (APCs) requiring expression of the transcription factors RORγt and PRDM16. Genetic perturbation of these APCs results not only in microbiota‐specific proinflammatory T cell responses but also in the breakdown of oral tolerance, which in turn predisposes to allergic inflammation. In this review, we summarize the discovery of these tolerance‐inducing APCs, highlight their role in instructing pTreg cell differentiation in response to microbiota and dietary antigens, and discuss the regulatory networks that support their function during intestinal immune tolerance.

## 1 Introduction

A fundamental challenge for the immune system is to distinguish between harmful pathogens and innocuous antigens derived from self, diet, or commensal microbes. To maintain homeostasis while preserving defense against infection, the adaptive immune system has evolved mechanisms not only to tolerate self‐antigens but also to actively suppress immune responses to non‐threatening antigens encountered in peripheral tissues. Central to this regulation are regulatory T (Treg) cells, which constrain excessive or inappropriate immune activation and thereby enforce immunological tolerance [1, 2]. Thymus‐derived Treg (tTreg) cells arise during T cell development through agonist selection mediated by AIRE‐expressing medullary thymic epithelial cells (mTECs), and are enriched for self‐reactive TCRs [3, 4, 5, 6, 7]. Although tTreg cells are critical for maintaining tolerance for self‐antigen in the periphery, and their deficiency results in a multitude of autoimmune manifestations, there is an additional layer of tolerance mediated by pTreg cells, which differentiate from naïve CD4^+^ T cells in response to antigen exposure within a tolerogenic milieu outside the thymus [8, 9]. pTreg cells play a particularly critical role at mucosal interfaces, principally the intestine, where the immune system is persistently challenged by abundant dietary and microbial antigens. There, pTreg cells help prevent excessive or misdirected immune responses from both T and B cells that could otherwise result in food intolerance and inflammatory bowel diseases.

Naïve T cells acquire diverse functions following their interactions with APCs that bridge innate and adaptive immunity. Innate stimuli in the tissue microenvironment, such as microbe‐derived TLR ligands, induce maturation of APCs, endowing them with the abilities to engulf and process antigens, enter the afferent lymphatics to migrate to secondary lymphoid organs and optimally present antigen‐MHC to T cells. Although multiple cell types express the MHC class II (MHCII) antigen presenting machinery, conventional dendritic cells (cDCs) have long been considered the “professional APCs” which drive naïve T cell priming and differentiation [10, 11]. Through engagement of both TCR and costimulatory ligands and secretion of cytokines, cDCs orchestrate T cell fate decisions. Historically, cDCs were thought to be plastic, capable of promoting both regulatory and effector T cell outcomes depending on their maturation state and local environmental cues [12, 13]. The concept of “tolerogenic dendritic cells” was proposed more than two decades ago, initially referring to immature DCs that were thought to promote peripheral tolerance under steady‐state conditions [14]. In this view, DCs were considered to exist along a maturation continuum, with immature DCs inducing T cell anergy, clonal deletion and Treg cell differentiation, and mature DCs driving effector and pro‐inflammatory responses. Subsequent studies then examined whether specific cDC subsets might possess an intrinsic capacity to promote pTreg cell differentiation. In vitro, CD103^+^ cDCs were observed to possess a superior ability to induce FOXP3^+^ Treg cells from naïve CD4^+^ T cells, relative to their CD103^−^ counterparts. This enhanced capacity was attributed to their higher expression of integrin α_v_β_8_, which activates latent TGF‐β, and retinaldehyde dehydrogenase RALDH2, which generates retinoic acid (RA)—both critical factors for Treg cell induction [15, 16, 17, 18, 19]. However, when exogenous active TGF‐β and RA were added to cultures, both CD103^+^ and CD103^−^ cDCs exhibited comparable Treg‐inducing activity [15, 16, 17, 18], suggesting that the functional differences between these subsets can be shaped by extrinsic cues, and that in vitro conditions may exaggerate their Treg‐inducing potential.

These observations with cultured primary cells raised the question of whether cDCs could similarly drive pTreg cell differentiation in vivo. Early studies showed that targeted delivery of antigens via anti–DEC‐205 conjugates led to T cell unresponsiveness upon rechallenge, supporting a role for DEC‐205^+^ DCs in inducing tolerance [20, 21]. Genetic studies offered mixed insights. Mice with *CD11c*‐*cre*–mediated deletion of integrin α_v_β_8_ or MHCII, as well as mice with *Zbtb46*‐*cre*‐mediated MHCII deletion, exhibited reduced pTreg frequencies and intestinal inflammation [17, 18, 22, 23, 24, 25]. In *Zbtb46*‐*DTR* bone marrow chimeric mice, treatment with diphtheria toxin (DT), which ablates both cDC1s and cDC2s, also led to a complete loss of food antigen‐specific pTreg cells and a breakdown of oral tolerance [26]. Because CD11c and ZBTB46 were widely used as markers of the cDC lineage, these findings were interpreted as indicating a potential role for cDCs in peripheral tolerance. *Zbtb46*‐*cre*;*Irf8* ^*fl*/*fl*^ mice, which lack cDC1s, exhibited only a partial defect in food antigen‐specific pTreg cell differentiation, while oral tolerance remained intact [26]. In addition, genetic ablation of cDC1s via deletion of the *Irf8* + 32 kb enhancer, as well as ablation of cDC2s using *CD11c*‐*cre*;*Irf4* ^*fl*/*fl*^ or *CD207*‐*DTA* mice did not alter microbiota‐ or food‐specific pTreg induction [27]. Together, these studies led to the prevailing view that peripheral tolerance could arise from functional redundancy among cDC subsets.

However, conditional deletion of MHCII using *Clec9a*‐*cre*, a cDC lineage driver that targets all cDC1s and a large fraction of cDC2s [28], had no impact on the generation of microbiota‐dependent or food antigen‐specific pTreg cells [29, 30, 31]. Moreover, when hematopoietic MHCII expression was restricted to *Clec9a*‐*cre*–expressing cells, food antigen‐specific pTreg cells failed to be induced [32]. These results argued that cDCs are neither required nor sufficient to induce pTreg cell differentiation, thereby refuting the notion of redundancy within the cDC compartment. This discrepancy can be explained by the fact that both *CD11c*‐*cre* and *Zbtb46*‐*cre* target broader hematopoietic cell populations beyond bona fide cDCs [33, 34], complicating the interpretation of earlier studies relying on these drivers. More recent work has demonstrated that intestinal pTreg cell differentiation is induced by a distinct lineage of APCs that express the transcription factor RORγt [29, 30, 31, 32, 35, 36, 37, 38]. These tolerance‐inducing APCs, which are targeted by both *CD11c*‐*cre* and *Zbtb46*‐*cre* but not by *Clec9a*‐*cre*, represent a previously unappreciated, specialized population responsible for maintaining peripheral tolerance at mucosal interfaces.

## 2 Discovery of RORγt ^+^ Tolerance‐Inducing APCs Mediating Intestinal Immune Homeostasis

RORγt is a nuclear hormone receptor expressed in double‐positive thymocytes, type 3 innate lymphoid cells (ILC3s, as well as lymphoid tissue inducer cells or LTi cells), and multiple subsets of peripheral T cells, orchestrating programs essential for thymic development, lymphoid organogenesis, and type 3 immunity [39, 40, 41, 42, 43, 44]. Recent studies have also identified a heterogeneous group of RORγt‐expressing pTreg‐inducing APCs, comprising MHCII^+^ ILC3s and various non‐ILC RORγt^+^ populations [45]. The definition and nomenclature of the non‐ILC subsets remain unsettled, encompassing tolerizing DCs (tolDCs) [36], RORγt^+^ extrathymic AIRE‐expressing cells (R‐eTACs or Janus cells) [38], Thetis cells (TCs) [30, 31], and RORγt^+^ DCs [37]. It is increasingly recognized that the non‐ILC populations are not mutually exclusive and are likely to overlap. Importantly, accumulating evidence suggests that ILC3s are not the APCs responsible for pTreg induction [31, 36, 37, 38].

ILC3s are often regarded as the innate counterparts of T helper 17 (Th17) cells and have well‐established roles in promoting intestinal homeostasis [46, 47]. During fetal development, LTi cells, which are often designated as ILC3s, but are derived from a PLZF^−^ progenitor distinct from PLZF^+^ ILC progenitors [48], induce the formation of secondary lymphoid organs [39, 40]. Post‐natally, LTi‐like cells induce tertiary lymphoid structures (TLSs) [49] and produce cytokines including IL‐22, GM‐CSF and IL‐17 in response to IL‐23 and IL‐1β [50, 51, 52], thereby supporting epithelial barrier integrity and host defense. Based on studies using *Rorc*(*t*)‐*cre*;*MHCII* ^*f*l/fl^ mice, ILC3s were initially proposed to suppress gut inflammatory T cell responses, restraining microbiota‐dependent Th17 cell differentiation without affecting the Treg compartment [53, 54]. However, this interpretation was complicated by two caveats. First, the conclusion that polyclonal Treg cells were unaffected was based solely on FOXP3 expression, which does not distinguish between tTreg and pTreg cells. This limited resolution likely obscured a selective effect on the pTreg population. Indeed, more recent studies demonstrated that *Rorc*(*t*)‐*cre*–mediated MHCII deletion leads to the loss of microbiota‐dependent RORγt^+^ pTreg cells [29, 30, 35]. Second, subsequent findings revealed that ILC3s are not the only RORγt^+^ non‐T cells [55, 56, 57]. Together, these findings indicated that *Rorc*(*t*)‐*cre*–driven manipulations affect a broader population of non‐ILC RORγt^+^ APCs than previously appreciated, thereby calling into question earlier interpretations and highlighting the need to explore the tolerogenic roles of these alternative APC subsets.

Among potential pTreg‐inducing APCs are RORγt^+^ cells that also express *Aire*. These eTACs were identified by single‐cell RNA sequencing of enriched innate immune cells pooled from lymph nodes of wild‐type mice, combined with GFP^+^ cells sorted from *Aire*‐*GFP* reporter mice [57]. Single‐cell transcriptomic analysis of tdTomato^+^MHCII^+^ innate immune cells isolated from the spleen and pooled lymph nodes of *Rorc*(*t*)‐*cre*;*Rosa26*‐*lsl*‐*tdTomato* fate‐mapped mice further defined three subsets of RORγt^+^ eTACs, termed R‐eTAC1 to R‐eTAC3, that were distinct from ILC3s [38]. Among these, *Aire* expression was higher in R‐eTAC3 and R‐eTAC1, whereas *Rorc* expression peaked in R‐eTAC2. Thetis cells were defined by single‐cell sequencing of MHCII^+^Venus^+^ innate immune cells isolated from the mesenteric lymph nodes (mLN) of *Rorc*‐*Venus*‐*creERT2* reporter mice and comprise four distinct subsets (TC I–IV), among which only TC I and TC III express *Aire* [30]. Index‐sorting analysis of cell‐surface markers showed that TCs form a phenotypic continuum ranging from CD11c^−/low^ (TC I) to CD11c^+^ (TC II–IV). In a separate study, single‐cell RNA sequencing was performed on CD11c^+^MHCII^+^tdTomato^+^ innate immune cells from the mLN of *Gm38411*‐*icre*‐*hCD2*;*Rosa26*‐*lsl*‐*tdTomato* fate‐mapped mice [37]. Because *Gm38411* expression closely mirrors that of *Rorc*, this strategy was used as an alternative to trace RORγt‐expressing populations. This analysis identified four clusters corresponding to the TC subsets, which were designated RORγt^+^ DC I–IV.

**FIGURE 1:**
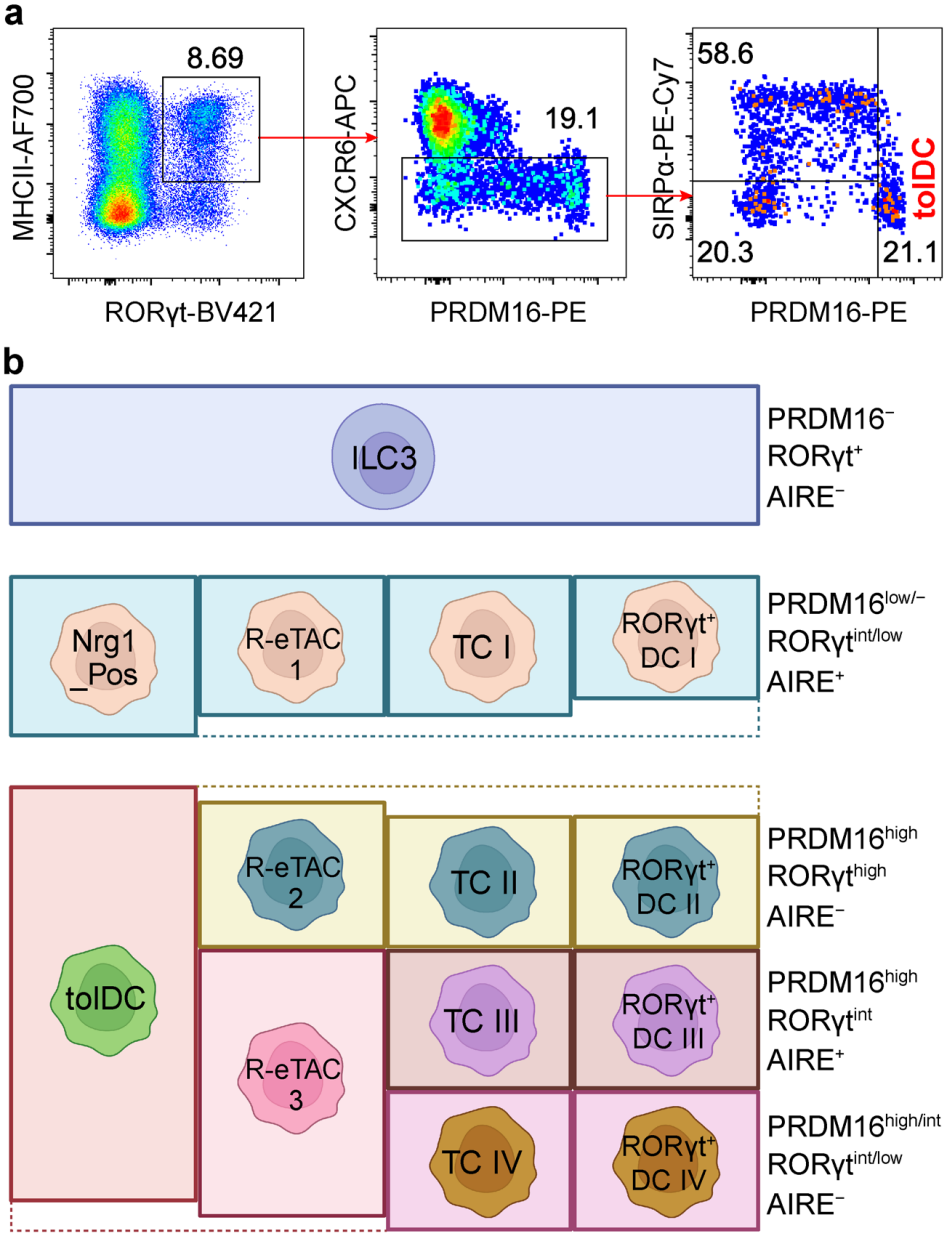
The phenotype of pTreg‐inducing RORγt^+^ APCs. (a) Gating strategy for tolDC. The left flow cytometry plot was gated on CD45^+^ (including CD45^low^) Ly6G^−^B220^−^TCRγδ^−^TCRβ^−^ cells and generated by concatenating mLN samples from four wild‐type mice. (b) Reconciling the family of RORγt^+^ APCs. Partial overlaps indicate that some of the cells in matched populations may not be shared. ILC3, type 3 innate lymphoid cell; R‐eTAC, RORγt^+^ extrathymic AIRE‐expressing cell; RORγt^+^ DC, RORγt^+^ dendritic cell; TC, Thetis cell; tolDC, tolerizing dendritic cell. Created in BioRender.

Using unbiased single‐cell RNA sequencing of all mLN MHCII^+^ innate immune cells, our group identified two *Rorc*‐expressing clusters distinct from both ILC3s and conventional cDC1 and cDC2 populations [36]. One of these clusters, termed Nrg1_Pos, most closely resembled TC I and was defined by its exclusive expression of Neuregulin 1 (*Nrg1*), a trophic factor involved in neuronal functions. The second cluster, named Prdm16_High, exhibited uniquely high expression of *Prdm16*, a PR‐domain–containing transcriptional regulator previously shown to have key functions in brown adipocytes, hematopoietic stem cells and cortical neurons [58, 59, 60, 61]. The Prdm16_High population expressed *Aire*, *Itgb8*, *Cd40*, *Ccr7*, and *Ly75* (which encodes DEC‐205), while lacking expression of *Cxcr6* and *Thy1* (*Cd90*), and exhibiting only minimal levels of *Il7r*. Importantly, the Prdm16_High cluster was selectively depleted in mice with *CD11c*‐*cre*–mediated RORγt inactivation—a strain shown to exhibit abrogated pTreg cell differentiation—suggesting that Prdm16_High APCs, rather than cDCs, ILC3s, or Nrg1_Pos cells, are the likely pTreg‐inducing APCs. This hypothesis was further supported by genetic evidence showing that conditional deletion of PRDM16 in RORγt‐expressing cells abrogated the generation of microbiota‐ and food antigen‐specific pTreg cells [36]. Single‐cell multiome profiling revealed that Prdm16_High APCs possess an epigenetic landscape more closely aligned with cDCs than with ILC3s. For example, although these cells lacked *Clec9a* transcription, the locus was nonetheless accessible, showing prominent chromatin peaks shared with cDCs but absent in ILC3s. Prdm16_High APCs could be clearly identified by flow cytometry as CD45^+^Ly6G^−^B220^−^TCRγδ^−^TCRβ^−^MHCII^+^RORγt^+^CXCR6^−^PRDM16^high^ cells in both the mLN and intestinal lamina propria. Inclusion of SIRPα in this panel further allows for clear discrimination of PRDM16^high^ cells (Figure 1a). It is possible that CXCR6^−^PRDM16^low/−^SIRPα^+^ RORγt^+^ APCs correspond to the Nrg1_Pos cluster, but the exact correspondence of these cells, as well as their function, remains unclear. Analysis with this gating strategy confirmed that Prdm16_High APCs were selectively lost when RORγt was inactivated in *CD11c*‐*cre* mice. Moreover, these cells were found to resemble cDCs in both surface phenotype and scatter characteristics, including cell size and granularity, while lacking expression of lymphoid lineage markers IL‐7R and CD90. Together, these findings define Prdm16_High APCs as a distinct RORγt^+^ population that closely resemble cDCs, yet uniquely induce pTreg cells specific for commensal and dietary antigens, supporting their designation as tolerizing dendritic cells (tolDCs) [36]. Putative human orthologs of tolDCs, defined by high co‐expression of *PRDM16* and *RORC*, as well as several other genes that are not expressed in cDCs or ILCs, have also been identified in multiple tissues, including mLN, intestines, tonsils, and spleen [36, 62, 63], indicating that these cells are evolutionarily conserved.

The evidence that RORγt^+^ cells distinct from ILC3 are pTreg‐inducing APCs is reinforced by results reported by several other groups. For example, inactivation of MHCII in *RORα*‐*cre* mice resulted in loss of MHCII in the vast majority of ILC3, but had no effect on gut pTreg frequencies [30]. A more selective approach, with *Serinc2*‐*icre*–mediated deletion of RORγt, which specifically depletes ILC3s, showed no impact on microbiota‐dependent pTreg generation [37]. In addition, *Rorc*(*t*)‐*cre*;*MHCII* ^*fl*/*fl*^ + *Il7r* ^*−*/*−*^ mixed bone marrow chimeras, which lack MHCII^+^ ILC3s, entirely rescued the generation of food antigen‐specific pTreg cells and restored oral tolerance [38]. These findings argue against a role for ILC3s in pTreg induction and further suggest that the relevant APCs develop and function independently of IL‐7R signaling.

The IL‐7R independence of pTreg‐inducing APCs suggests a non‐lymphoid origin, consistent with our findings that tolDCs share phenotypic and epigenetic features with cDCs [36]. Nonetheless, RORγt^+^ DCs were reported to likely originate from lymphoid progenitors, although this conclusion was primarily based on in vitro culture systems [37]. Thus, the ontogeny of pTreg‐inducing APCs remains a key unresolved question, and the precise progenitor populations and transcriptional programs that govern their specification are yet to be defined. Analysis of tolDC number in the mouse mLN revealed a rapid increase in early postnatal life, leveling off after weaning into adulthood [36], whereas total TCs and ITGβ8^+^ TC IV were reported to drop off in numbers in the gut‐draining lymph nodes after around 2–3 weeks of age [30, 31]. Notably, both tolDCs and TCs in mLN were found to be most abundant in proportion during the first postnatal week and progressively declined thereafter [30, 36], a pattern that may underlie the reduced efficiency of pTreg induction by oral antigens in adult mice compared to 12‐day‐old neonates [31]. These results are consistent with the requirement for early postnatal establishment of tolerance to newly encountered intestinal antigens and the observation that adult mice can still develop tolerance to commensal and dietary antigens [26, 30, 31, 35, 36, 64], indicating that pTreg‐inducing RORγt^+^ APCs remain functionally competent beyond early life. Further studies are needed to determine their turnover and whether there is a dropoff in these APCs with aging.

## 3 Reconciling the Multiple Designations of pTreg‐Inducing RORγt ^+^ APCs

The emergence of multiple designations for non‐ILC RORγt^+^ populations has complicated the comparison and integration of findings across studies. A precise and standardized definition of the pTreg‐inducing APC subset is therefore needed. For instance, *Aire* expression was observed in only a fraction of the tolDC and Nrg1_Pos populations [36], indicating that tolDCs cannot be classified as AIRE‐expressing R‐eTACs. Likewise, CD11c expression was detected in a subset of tolDCs [36], suggesting that they cannot be subsumed within TC II–IV or RORγt^+^ DC II–IV, both of which exhibited CD11c expression [30, 37]. Since there was complete loss of commensal microbe‐specific pTreg induction in mice with *CD11c*‐*cre*–mediated inactivation of MHCII, CCR7, or integrin α_v_, this observation implies that CD11c may be partially downregulated during tolDC lineage progression. To reconcile the diverse definitions of RORγt^+^ APCs, we sought to integrate multiple nomenclatures [36, 37, 38, 65] (Figure 1b). Briefly, Nrg1_Pos cells largely correspond to TC I, RORγt^+^ DC I and R‐eTAC1, whereas tolDCs show substantial overlap with TC II–IV, RORγt^+^ DC II–IV and R‐eTAC2/3. Although one study reported that DT‐mediated ablation of AIRE^+^ cells in *Aire*‐*DTR* mice led to a partial reduction in food antigen‐specific pTreg cells [38], other studies challenged the functional relevance of AIRE‐expressing RORγt^+^ APCs in intestinal pTreg cell induction [31, 35]. Specifically, ablation of eTACs in *Aire*‐*DTR* bone marrow chimeric mice using DT [35], or genetic deletion of R‐eTACs using *Rorc*(*t*)‐*cre*;*Aire* ^*flex*‐*dtA*^ mice [31], did not affect the differentiation of microbiota‐ and food‐specific pTreg cells, respectively. Because TC IV express the highest levels of ITGβ8, they were proposed to be the exclusive pTreg‐inducing APCs [30, 31]. However, a key caveat is that TC II and TC III also express ITGβ8, albeit at lower levels, and show chromatin accessibility at the *Itgb8* locus resembling TC IV [30]. Moreover, although TC II display the highest level of *Runx3* expression [30], germline attenuation of *Runx3* caused a severe reduction across TC II–IV subsets and in endogenous RORγt^+^ pTreg cells [66]. This illustrates that higher expression of a key gene in one subset does not preclude other subsets sharing the same function, especially given that the expression of such critical genes may be dynamic rather than sustained. It also remains possible that functional redundancy exists among tolDC subsets, such that ablation of one subset may be compensated by others. Therefore, while tolDCs are required for pTreg induction, it remains unclear whether all tolDC subsets are equally essential, or whether this function is restricted to a specific fraction within the broader tolDC compartment. In light of this uncertainty, and because the term “tolDC” is functionally and genetically grounded, defined by dependence on the essential transcription factors PRDM16 and RORγt, we adopted tolDC as the working designation. Future work should clarify relationships among tolDC subsets and develop subset‐specific genetic tools to test the necessity and sufficiency of well‐defined APCs for pTreg induction in vivo.

## 4 Transcriptional Regulation of tolDCs

Multiple transcription factors have been identified in tolDCs, including PRDM16, RORγt, RUNX3, ZBTB46, IRF8 and AIRE. Among these, PRDM16 and RORγt have emerged as essential regulators, as genetic ablation of either gene in tolDCs results in a failure to induce pTreg cells and mucosal tolerance [36]. Notably, while RORγt is required for tolDC development, as evidenced by the complete loss of the tolDC cluster in *CD11c*‐*cre*;*Rorc*(*t*)^*fl*/*gfp*^ mice based on single‐cell transcriptomic and flow cytometry analyses, it remains unclear whether PRDM16 affects tolDC development or primarily regulates their function. A similar single‐cell analysis in *Rorc*(*t*)‐*cre*;*Prdm16* ^*fl*/*fl*^ mice will be needed to address this question. Moreover, the transcriptional program orchestrated by PRDM16 and RORγt that underlies tolDC development or function remains unclear. Through systematic dissection of *Rorc*(*t*) cis‐regulatory elements, a previously uncharacterized enhancer, *Rorc*(*t*) + 7 kb, was found to be chromatin‐accessible in both tolDCs and ILC3s and to regulate RORγt expression [36]. Mouse models lacking the *Rorc*(*t*) + 7 kb enhancer displayed reduced numbers of both tolDCs and RORγt‐expressing ILC3s. Although ILC3/LTi‐dependent functions such as lymphoid organ development and resistance to *Citrobacter rodentium* remained intact, these mice exhibited a marked reduction in endogenous RORγt^+^ pTreg cells and an abrogation of microbiota‐ and food‐specific pTreg cell differentiation [36, 37, 67]. This indicates that the residual tolDC‐like cells lacking the enhancer are either functionally compromised or constitute a distinct cell state or lineage not dependent on the *Rorc*(*t*) + 7 kb enhancer. The *Rorc*(*t*) + 7 kb enhancer is highly conserved between mouse and human [68], harbors multiple RUNX3‐binding motifs [37, 67], and is accessible in tolDCs from both species [36]. Consistent with this, a recent study showed that attenuation of RUNX3/CBFβ complexes through germline mutation resulted in profound defects in tolDC development and significant reduction of intestinal pTreg cells [66].

Although ZBTB46 is expressed in tolDCs [30, 36], there is currently no direct evidence for a functional requirement of ZBTB46 in tolDC biology. One study reported that deletion of ZBTB46 in *Rorc*(*t*)‐*cre*–expressing cells resulted in an increase in intestinal Th17 cells, although changes in pTreg cells were not examined [34]. While this observation raises the possibility that ZBTB46 might influence tolDC function, further investigation is needed. Insights into the potential role of IRF8 in tolDCs come from a recent study reporting that deletion of IRF8 in RORγt^+^ cells led to reduced induction of adoptively‐transferred food‐specific pTreg cells, while having a modest effect on endogenous RORγt^+^ pTreg cells [31]. scRNAseq analysis in this study revealed high *Irf8* expression in TCIV, with lower levels in TCII and TCIII. However, the analysis was limited to RORγt^+^ APCs, and broader comparisons with cDC subsets revealed that RORγt^+^ DCs express substantially lower levels of *Irf8* relative to cDC1s [37]. Furthermore, a previous study showed that oral tolerance remained intact in *Zbtb46*‐*cre*;*Irf8* ^*fl*/*fl*^ mice [26], which are now recognized to also delete IRF8 in tolDCs. These observations appear inconsistent regarding a role for IRF8 in tolDC function and underscore the need for further investigation using complementary models for pTreg induction by dietary antigen and microbiota. In contrast, AIRE is considered dispensable for tolDC‐mediated pTreg generation in response to both microbial and dietary antigens [29, 35, 38]. Recent studies showed that deletion of AIRE, either in RORγt^+^ cells or during early hematopoiesis using *Vav1*‐*icre*, did not affect the induction of microbiota‐ or food‐specific pTreg cells [35, 38]. While AIRE is well known to promote ectopic expression of tissue‐restricted antigens [69, 70, 71], whether it contributes to the generation of self‐antigen–specific pTreg cells remains unclear, as suggested by a recent report implicating cDCs in this process [72]. Further studies are needed to determine whether tolDCs contribute to self‐tolerance.

## 5 tolDCs in Microbiota Tolerance

The intestinal microbiota forms a dense and diverse ecosystem that coexists with the host in a largely mutualistic relationship. Commensal microbes are essential for numerous physiological processes, including digestion, nutrient synthesis, epithelial barrier maintenance, and immune regulation [73, 74, 75, 76]. However, not all host–microbiota interactions are uniformly beneficial. Under certain environmental or genetic conditions, specific members of the microbiota can reveal their pathogenic potential, eliciting immune activation and driving chronic inflammation [77, 78]. These conditionally pathogenic organisms, termed pathobionts, include species such as *Helicobacter hepaticus* (*Hh*), which persistently colonize the murine large intestine and are capable of inducing either tolerogenic or proinflammatory immune responses depending on the local context [64, 79, 80, 81, 82]. The microbiota‐dependent RORγt^+^ pTreg cells have been implicated in promoting tolerance to commensal bacteria. These cells are substantially reduced in germ‐free or antibiotic‐treated mice, and deletion of RORγt in FOXP3‐expressing cells exacerbates intestinal inflammation in multiple colitis models, underscoring their critical role in maintaining intestinal immune homeostasis [43, 83, 84]. We previously demonstrated that naïve *Hh*‐specific CD4^+^ T cells differentiate into RORγt^+^ Treg cells, in a process dependent on intrinsic c‐MAF expression [64]. Although RORγt deficiency in Treg cells had only a modest effect on the *Hh*‐specific pTreg and Th17 cell balance, c‐MAF inactivation severely impaired *Hh*‐specific pTreg cell differentiation, compromised IL‐10 production, and resulted in the expansion of inflammatory *Hh*‐specific Th17 cells and spontaneous colitis [64]. These findings suggested that pathobiont‐driven intestinal inflammation could arise from a failure to generate functional microbiota‐specific Treg cells.

Results indicating that one commensal microbe could induce the differentiation of multiple functional subsets of CD4^+^ T cells raised the question of which APCs are responsible for instructing each microbiota‐specific T cell program. Recent findings suggest that there are distinct APCs that program pTreg, pathogenic Th17 (pTh17, distinct from regulatory‐like homeostatic Th17 cells [85]) and T follicular helper (Tfh) cells in response to *Hh* [35]. For *Hh*‐specific T cell differentiation, RORγt^+^ APCs, likely tolDCs, were both necessary and sufficient for inducing the pTreg program. Thus, inactivation of MHCII in *Rorc*(*t*)‐*cre*–expressing cells abolished *Hh*‐specific pTreg cells and led to a marked reduction in endogenous RORγt^+^ pTreg cells [29, 30, 35], while expression of MHCII exclusively in *Rorc*(*t*)‐*cre*–expressing bone marrow‐derived APCs restored solely pTreg cells [35]. In contrast, expression of MHCII in *CD11c*‐*cre*‐expressing APCs resulted in the differentiation of both pTreg and Tfh cells specific for *Hh*, consistent with cDCs instructing the Tfh program [35]. Remarkably, loss of MHCII in *CD11c*‐*cre*‐expressing cells resulted not only in the loss of Tregs, but also in the differentiation of *Hh*‐specific pTh17 cells. The precise identity of cells that take over the priming and programming of pTh17 cell differentiation in the absence of pTregs is unknown at this time, although monocyte‐derived cells are likely candidates [86, 87, 88]. Although such myeloid cells may be required to direct the differentiation of pTh17 cells under all circumstances, it is possible that they are engaged only when required to compensate for the absence of cDCs that normally have such a function.

Use of *Rorc*(*t*)‐*cre* mice allowed for further dissection of APC requirements in pTreg cell induction. Deletion of *H2*‐*DMa*, encoding a molecule essential for processed peptide loading onto MHCII [89, 90, 91], confirmed that the antigen‐processing machinery is required in these cells, ruling out an unconventional function for MHCII. Genetically knocking out either subunit of integrin α_v_β_8_ similarly resulted in loss of pTreg induction. Experiments with mixed bone marrow chimeric mice demonstrated that MHCII and integrin α_v_β_8_ must be expressed by the same APC for pTreg cell differentiation, consistent with the notion that the same cell performs both antigen presentation, presumably priming naïve T cells, and instruction of the differentiation pathway, through activation of latent TGF‐β [30, 35]. Inactivation of CCR7, using *Rorc*(*t*)‐*cre*, *CD11c*‐*cre* or *Zbtb46*‐*cre* mice, similarly abrogated *Hh*‐specific pTreg cell generation [35]. This may be due to a requirement for CCR7‐mediated migration of tolDC through afferent lymphatics from the lamina propria to the mLN and/or within different regions of the mLN.

**FIGURE 2:**
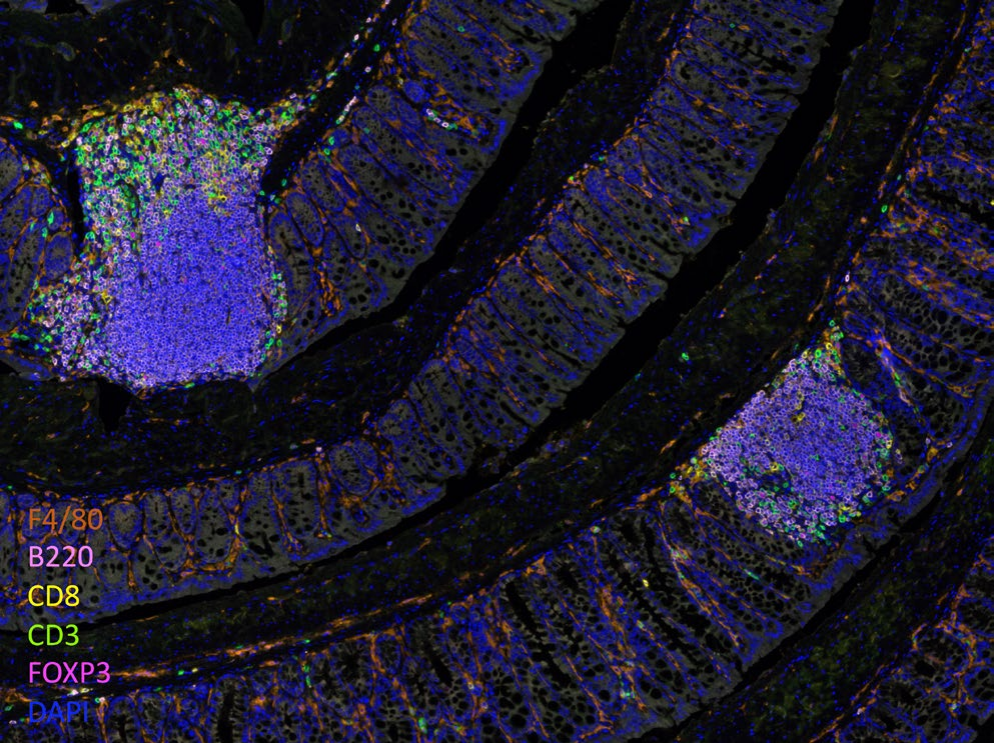
Formation of tertiary lymphoid structures in the large intestine of *Helicobacter hepaticus* ‐colonized *CD11c*‐*cre*;*Ccr7* ^*fl*/−^ mice. Images were generated with Opal platform. F4/80 (orange), B220 (pink), CD8 (yellow), CD3 (green), FOXP3 (magenta), and DAPI (blue).

All *Hh*‐colonized mutant mice with loss of pTreg cells due to defective tolDCs displayed marked expansion of RORγt‐ and T‐bet‐expressing *Hh*‐specific pTh17 cells [29, 35, 36]. Notably, germ line inactivation of *Ccr7* also resulted in the loss of pTreg cells and the expansion of pTh17 cells. Thus, priming and differentiation of pathogenic Th17 cells can be mediated by APCs that do not express CD11c and are independent of CCR7. How such APCs, presumably monocyte‐derived, acquire antigen and localize to the mLN are important unanswered questions. When CCR7 loss was confined to CD11c^+^ cells in *Hh*‐colonized mice, the resulting large intestine inflammation was accompanied by formation of well‐organized TLSs (Figure 2). TLSs were not present when T cells were additionally deprived of CCR7 (in germ line *Ccr7* mutants and *Rorc*(*t*)‐*cre* conditional mutant mice), reflecting defective migration of naïve T cells to the mLN, but it is unclear whether pro‐inflammatory pTh17 cells are the instigators of the ectopic lymphoid tissues. It should be noted that all of these studies were conducted in the context of *Hh* colonization, which contributes substantially to colitogenesis, and it remains possible that other pathobionts direct inflammatory T cell differentiation through divergent mechanisms. In addition, colorectal tumor‐associated TLSs that promote B cell‐dependent anti‐tumor immunity dependent on *Hh*‐specific Tfh cells were induced following *Hh* colonization [92]. How formation of such pathobiont‐dependent TLSs differs under conditions of IL‐10 deprivation versus tumor association may provide important insights into differences between pathogenic versus anti‐tumor functions of these structures.

## 6 tolDCs in Food Tolerance

In addition to maintaining immune tolerance to commensal microbiota, the immune system must also avoid inappropriate activation in response to harmless dietary antigens. This is achieved through oral tolerance, a fundamental immunological mechanism that restrains excessive immune responses both locally in the gut and systemically [93, 94]. Failure to establish oral tolerance can result in food allergy, which is marked by aberrant immune responses to dietary proteins and may manifest as intestinal inflammation, diarrhea, or even life‐threatening anaphylaxis [95, 96]. pTreg cells play a central role in the establishment of oral tolerance [97, 98, 99]. Mice lacking the *Foxp3* enhancer CNS1, an element that contributes to TGF‐β–dependent pTreg cell differentiation but is largely dispensable for tTreg cell development [100], display spontaneous intestinal inflammation characterized by plasmacytic enteritis and elevated expression of Th2 cytokines in CD4^+^ T cells [101]. Building on their role in microbiota tolerance, tolDCs have emerged as key APCs for the induction of pTreg cells in response to food antigens. Mice with selective depletion of tolDCs failed to generate both RORγt^+^ and RORγt^−^ intestinal pTreg cells specific for orally administered ovalbumin (OVA) [36]. Deletion of MHCII or integrin α_v_β_8_ in RORγt^+^ cells likewise abolished this response [31, 32, 36, 38], whereas restricting hematopoietic MHCII expression to *Rorc*(*t*)‐*cre*–expressing cells restored OVA‐specific pTreg cell differentiation [32]. These observations suggest that RORγt^+^ APCs, probably tolDCs, function as both the required and sufficient APC population for the induction of food antigen‐specific pTreg cells.

**FIGURE 3:**
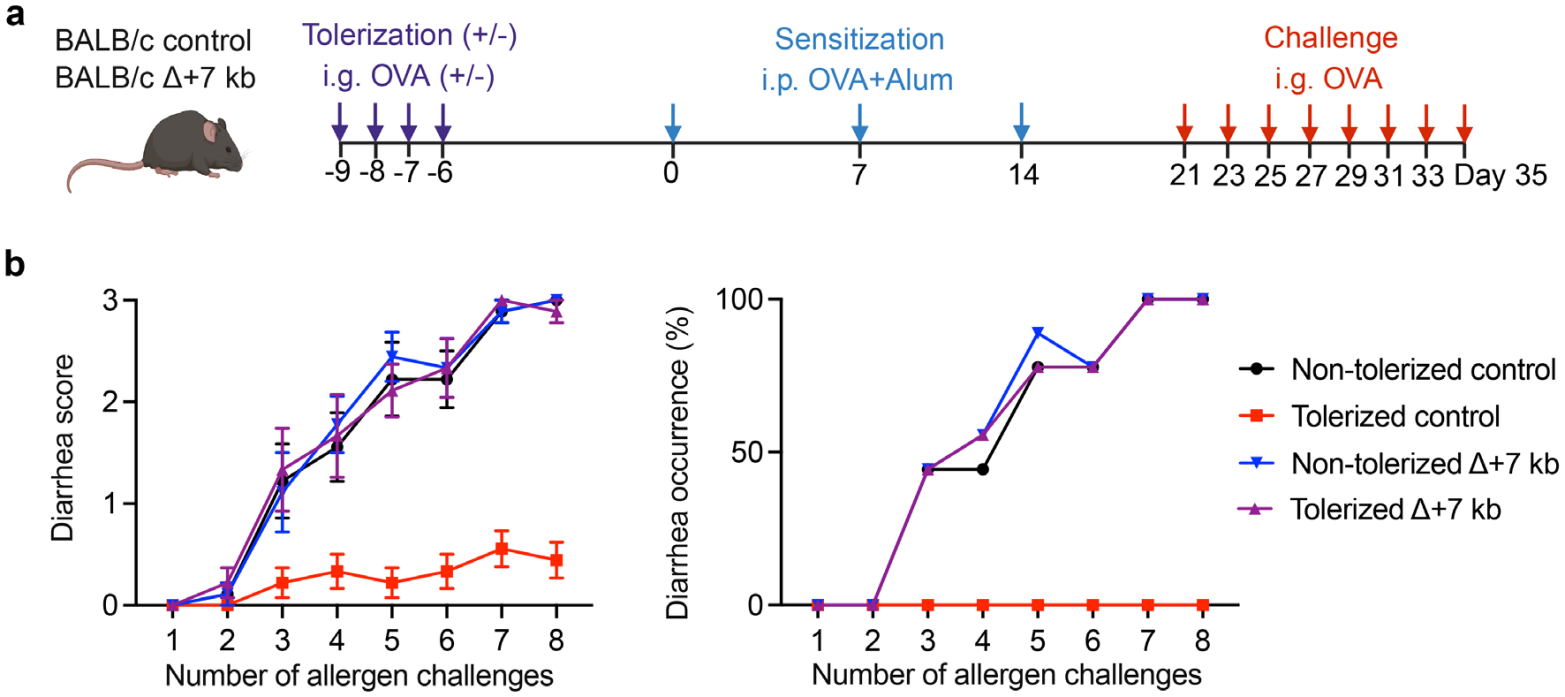
tolDCs are required for developing oral tolerance against food allergy. (a) Experimental design for the food allergy experiments in (b). Created in BioRender; control, *Rorc*(*t*) + 7 kb^+/−^; *Δ* + 7 kb, *Rorc*(*t*) + 7 kb^−/−^; i.g., intragastric (50 mg of OVA); i.p., intraperitoneal (100 μg of OVA mixed with 1 mg of Alum). All mice were on the BALB/c background. Mice were fasted for 4 h prior to each intragastric challenge. (b) Diarrhea was assessed 1 h after the intragastric challenge by visually monitoring mice for up to 30 min. Stool consistency was scored as follows: 0, normal stool; 1, soft stool; 2, loose stool; 3, watery stool. Mice exhibiting loose or watery stool were recorded as diarrhea‐positive. *n* = 9 per group. Data are pooled from two independent experiments. Data are mean ± s.e.m.

Dysfunction of tolDCs results in heightened effector T helper cell responses to food antigens, although the specific subset of T helper cells that dominates may vary depending on the local tissue microenvironment [31, 32, 36, 37, 38, 67]. Notably, three studies reported enhanced Th2 responses and the subsequent development of spontaneous type 2 gastrointestinal pathology in aged C57BL/6 mice lacking functional tolDCs [36, 37, 67], but there was variable expansion of Th17 cells, suggesting that environmental conditions within different animal facilities may influence susceptibility to type 2 responses. When these mice were subjected to adjuvant‐based sensitization following OVA tolerization, oral tolerance was disrupted; however, the nature of the immune response elicited upon subsequent OVA challenge depended on the type of adjuvant used. For example, tolDC dysfunction resulted in robust Th2 responses and IgE production in the context of alum‐induced allergic airway inflammation [31, 36, 37], whereas in a complete Freund's adjuvant (CFA)‐induced delayed‐type hypersensitivity model it led to strong IgG2c responses [38]. Using a cholera toxin‐induced food allergy model, we further demonstrated that tolerized tolDC‐deficient mice failed to be protected from IgE‐mediated anaphylactic responses upon systemic OVA challenge, as evidenced by a rapid decline in core body temperature [36]. It is important to note that these studies were performed in C57BL/6 mice, a strain known to exhibit resistance to overt allergic symptoms following oral antigen exposure [102, 103]. To better recapitulate clinically relevant features of food allergy, we used CRISPR‐mediated genome editing to generate *Rorc*(*t*) + 7 kb knockout BALB/c mice, which are more susceptible to food antigen‐induced allergic responses. Following OVA tolerization, these mice were subjected to a food allergy model involving direct intragastric OVA challenge. In this experimental setting, only tolerized control littermates, but not tolDC‐deficient BALB/c *Rorc*(*t*) + 7 kb knockout mice, were protected from diarrhea induced by oral OVA challenge (Figure 3), highlighting the essential role of tolDCs in developing oral tolerance to dietary antigens.

## 7 Cellular Networks Mediating tolDC‐Induced pTreg Function

The identification of tolDCs supports the emerging concept that certain APC lineages are dedicated to driving distinct T cell differentiation programs [104]. As a case in point, tolDCs function as specialized inducers of microbiota‐ and food antigen‐specific pTreg cells [36]. Yet, how T cell responses to these antigens are instructed in the absence of functional tolDCs remains incompletely understood. In general, recognition of conserved microbial structures by pattern recognition receptors (PRRs) triggers the initiation of antimicrobial Th1 and Th17 responses [105]. Consistently, MyD88 signaling in myeloid cells is required to initiate inflammatory T cell responses and colitis induced by pathobionts such as *Hh* in the absence of IL‐10 signaling [106, 107, 108]. *Hh*‐induced chronic intestinal inflammation also depends on IL‐23 production by CD11c^+^ myeloid cells following MyD88 activation [81, 109, 110, 111]. Notably, MHCII expression on CD11c^+^ cells is dispensable for the generation of *Hh*‐specific pTh17 cells [35], indicating that their critical role lies in providing cytokine signals for pTh17 differentiation, even if other APCs can perform the T cell priming function. The persistence of *Hh*‐specific pTh17 cells in CCR7‐deficient mice further suggests that the APCs mediating their priming can operate independently of CCR7 [35]. Further investigation is needed to delineate the precise identity of these pTh17‐inducing APCs.

Spontaneous inflammation triggered by food proteins is typically mediated through Th2‐driven allergic responses. The mechanisms initiating type 2 immunity remain incompletely understood, although tissue perturbations are thought to play a role [112, 113]. A recent study provided important insight by showing that common mold allergens can initiate type 2 responses through the formation of transmembrane pores in airway epithelial cells [114]. However, the mechanisms by which food allergens, which are not intrinsically immunogenic, initiate type 2 immunity remain unclear. Early studies using antibodies against FcɛRI, the high‐affinity IgE receptor, suggested that basophils could act as APCs for allergen‐ and helminth‐induced Th2 responses [115, 116, 117]. However, genetic ablation of basophils did not impair Th2 responses to helminth infection [118, 119]. These discrepancies may reflect differences in depletion efficiency and specificity. Subsequent studies identified IRF4‐ and KLF4‐dependent cDC2s as critical for supporting Th2 responses to airway and skin allergens and to helminths [120, 121, 122, 123, 124]. Nevertheless, restricting MHCII expression to CD11c^+^ cells was insufficient to promote alum‐induced allergic airway inflammation or to elicit protective Th2 immunity against intestinal helminth infection [115, 125]. Thus, the antigen‐presenting cells responsible for initiating Th2 priming remain to be clearly defined.

**FIGURE 4:**
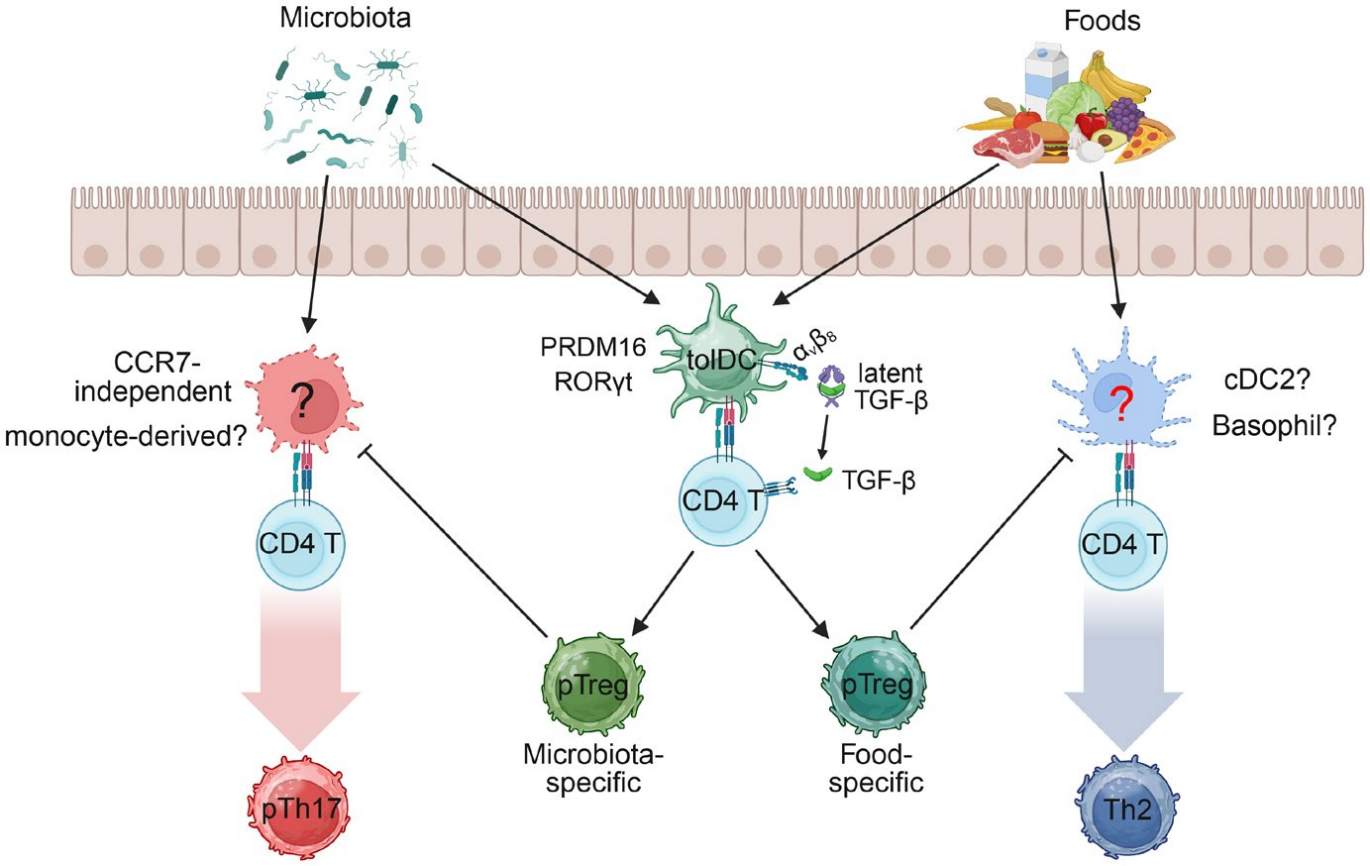
Schematic model of tolDC‐mediated regulation of intestinal T cell responses. Microbiota‐ and food‐derived antigens are sampled in the intestine and presented to CD4^+^ T cells by specialized antigen‐presenting cells in the mesenteric lymph nodes (mLN). PRDM16‐ and RORγt‐dependent tolerizing dendritic cells (tolDCs) induce peripheral regulatory T (pTreg) cell differentiation by engaging naïve or antigen‐experienced CD4^+^ T cells and activating latent TGF‐β via integrin α_v_β_8_. In the absence of tolDC‐induced antigen‐specific pTreg cells, CCR7‐independent APCs of undefined identity, potentially monocyte‐derived, drive microbiota‐specific pathogenic Th17 (pTh17) cell differentiation. The identity of APCs responsible for instructing food antigen‐specific Th2 responses also remains uncertain, but conventional type 2 dendritic cells (cDC2s) and basophils are potential candidates. Created in BioRender.

The mechanisms by which tolDC‐induced pTreg cells maintain intestinal homeostasis are still incompletely understood. Treg cells suppress immune responses through multiple mechanisms, including contact‐dependent inhibition of APCs, secretion of anti‐inflammatory cytokines, IL‐2 consumption, adenosine generation, and, in certain contexts, cytotoxic activity [126]. Antigen specificity appears to be particularly important for intestinal Treg cell function, as epitope‐specific pTreg cells are more effective at suppressing *Hh*‐induced colitis [64]. The persistence of abundant endogenous RORγt^−^ Treg cells in tolDC‐deficient mice, which nevertheless fail to control inflammatory T cell responses, supports this notion [36]. Consistent with this, TCR expression is critical for the suppressive function of differentiated Treg cells [127, 128], although this requirement could also reflect the need for tonic TCR signaling for Treg maintenance. Treg‐mediated suppression of cytotoxic T lymphocytes has been shown to depend on MHCII‐dependent interactions with cDC1s [129], in which CTLA‐4‐driven removal of the costimulatory molecules CD80 and CD86 may play a central role [130, 131, 132, 133]. Using a proximity‐labeling strategy (LIPSTIC), a recent study demonstrated that cDCs can present dietary antigens to CD4^+^ T cells in mLN under tolerizing conditions [134]. This study also showed that cDCs partially contribute to the initial priming and induction of food antigen‐specific pTreg cells, although another report failed to observe such a function [31]. A separate study offered an alternative interpretation, suggesting that pTreg–cDC1 interactions during homeostasis function primarily to limit the expansion of food‐specific CD8αβ T cells [32]. Together, current evidence supports a working model in which antigen‐specific suppression of APCs that drive inflammatory T cell responses represents a critical mechanism by which tolDC‐induced pTreg cells sustain intestinal homeostasis (Figure 4). cDCs may additionally have an important role in either priming naïve cells destined to become pTregs or in maintaining or expanding differentiated pTregs. Even though tolDCs can function in isolation to perform both priming of naïve T cells and instruction of the pTreg program, this may be due to compensation in the absence of cDCs. Further genetic and spatial studies will be necessary to fully characterize the mechanism of pTreg cell induction.

## 8 Perspectives and Future Directions

Although tolDCs are now recognized as specialized inducers of pTreg cells in response to commensal microbiota and dietary antigens, many important questions remain. A central question is what distinguishes tolDCs from other APCs, such that only tolDCs can induce pTreg cell differentiation in vivo. Despite their rarity, tolDCs possess exceptional tolerogenic capacity. This raises the question of how much of their unique function derives from having a distinct transcriptional program rather than their residence in specialized anatomical niches optimized for tolerogenic priming. The transcriptional program can be explored by dissecting the regulatory network governed by key regulators such as PRDM16, RORγt, and RUNX3. Resolving tolDC spatial localization and functional context will require integrated approaches such as high‐resolution imaging and spatial transcriptomics combined with genetic perturbation models. The migratory trajectory of naïve CD4^+^ T cells within the mLN also remains to be elucidated, including where and how they encounter distinct APC subsets. A hypothesis that remains to be tested is that pTreg induction may occur through a two‐step process, involving initial priming by more generic APCs, followed by a secondary encounter with tolDCs that recognize the same antigen to drive FOXP3^+^ T cell differentiation. Furthermore, detailed functional studies have so far been confined to a narrow set of experimental models, focusing primarily on *Hh* as a representative commensal bacterium and OVA as a model dietary antigen, leaving the role of tolDCs in tolerance to other microbial species and diverse food antigens to be elucidated. In addition to canonical Foxp3^+^ pTreg cells, other peripherally generated regulatory T cell subsets, including IL‐10‐producing Foxp3^−^ T regulatory type 1 (Tr1) cells and intraepithelial CD4^+^ T cells (CD4_IELs_), have been shown to contribute to mucosal tolerance [135, 136, 137, 138, 139, 140]. The APCs that drive the differentiation of these alternative regulatory populations remain to be identified.

The efficiency of peripheral tolerance induction is likely highest early in life, when tolDCs encounter little competition from other APCs and play a dominant role in establishing immune tolerance to newly encountered commensal and dietary antigens. This early bias may represent an adaptive strategy to ensure that such tolerance is established before strong sensitization or immune activation occurs. In adulthood, exposure to new commensal and dietary antigens becomes less frequent, and although the DC compartment may gradually shift toward more protective programs that support pathogen defense, tolDCs remain functionally active and continue to maintain immune homeostasis under steady‐state conditions.

Whether tolDCs are present in tissues beyond the mLN and intestine and whether they mediate tolerance to other classes of antigens, such as tumor neoantigens and alloantigens, is still unclear. Using *Irf8* + 32 kb knockout mice, which lack cDC1s, two recent studies reported that cDC1s were required for costimulation blockade–mediated long‐term cardiac allograft acceptance [141, 142]. It will be important to determine whether tolDCs also contribute to this process. In addition, the mechanisms underlying antigen acquisition by tolDCs in vivo also remain to be fully elucidated, particularly whether antigens are captured directly by tolDCs or transferred from other cells, as has been reported for CX3CR1^+^ macrophages transferring antigen to cDCs [143]. The development of genetic tools that allow for selective targeting of tolDCs in vivo without affecting other cell types will be critical for further dissecting their biology. Such tools would not only clarify their physiological role but also enable translational exploration. For example, one could envision therapeutically targeting tolDCs to enforce antigen‐specific tolerance by inducible expression or targeted delivery of candidate antigens in both prophylactic and therapeutic settings. However, such approaches will require a deeper understanding of whether and how environmental factors such as inflammation and the microbiota influence tolDC function and stability. By enforcing immune tolerance, tolDCs offer a promising therapeutic avenue for treating allergic disorders, inflammatory bowel diseases, autoimmune diseases, and transplant rejection.

## Conflicts of Interest

D.R.L. is cofounder of Vedanta Biosciences and ImmunAI, on the advisory boards of IMIDomics, Sonoma Biotherapeutics, NILO Therapeutics, and Evommune, and on the board of directors of Pfizer Inc. L.F. declares no competing interests.

## Data Availability

The data that support the findings of this study are available on request from the corresponding author.
